# Reducing Birth Defects by Decreasing the Prevalence of Maternal Chronic Diseases—Evaluated by Linked National Registration Dataset

**DOI:** 10.3390/children9121793

**Published:** 2022-11-22

**Authors:** Lih-Ju Chen, Ping-Ju Chen, Jing-Yang Huang, Shun-Fa Yang, Jia-Yuh Chen

**Affiliations:** 1 Institute of Medicine, Chung Shan Medical University, Taichung 40201, Taiwan; 2 Division of Neonatology, Changhua Christian Children’s Hospital, Changhua 50050, Taiwan; 3 Department of Post-Baccalaureate Medicine, College of Medicine, National Chung Hsing University, Taichung 40227, Taiwan; 4 Department of Dentistry, Changhua Christian Hospital, Changhua 50050, Taiwan; 5 Department of Medical Research, Chung Shan Medical University Hospital, Taichung 40201, Taiwan

**Keywords:** birth defect, maternal chronic disease, prevalence, population attributable risk percent

## Abstract

Birth defects (BDs) are an important cause of abortion, stillbirth, and infant mortality that may cause lifelong disability. The defects can be caused by genetics, environmental exposure, or maternal chronic diseases. We conducted a study to analyze the association between maternal chronic diseases and BDs and to evaluate the effect of decreasing the prevalence of maternal chronic diseases on reducing BDs. The data of newborns and their mothers were concatenated and analyzed from three national population databases: the National Health Insurance Research Database, the Birth Certificate Application, and the Birth Registration Database in Taiwan during the period of 2005 to 2014. Codes 740-759 of the International Classification of Diseases 9th Revision—Clinical Modification (ICD-9-CM) were used as the diagnosis of BDs. The prevalence of BDs was 2.72%. Mothers with cardiovascular diseases, hypertension, anemia, genitourinary tract infections, renal diseases, neurotic or psychotic disorders, gestational diabetes mellitus (DM), and pregestational type 1 or type 2 DM had a significantly higher prevalence of BDs. The population attributable risk percent (PAR%) of BDs was 1.63%, 0.55%, 0.18%, 1.06%, 0.45%, 0.22%, 0.48%, and 0.24% for maternal hypertension, cardiovascular disease, renal disease, genitourinary infection, anemia, neurotic and psychotic disorders, gestational DM, and pregestational type 1 or type 2 DM, respectively. The percentage change (−1%, −5%, and −10% of prevalence in 2034 compared with the prevalence in 2005–2014) of maternal disease and the predicted number of live births was used to estimate the decrease in the number of newly diagnosed BDs in 2034. By using the middle-estimated number of live births in 2034, we predicted that the number of BDs would decrease by 302, 102, 33, 196, 83, 41, 89, and 44 with a −5% prevalence of maternal hypertension, cardiovascular disease, renal disease, genitourinary infection, anemia, neurotic and psychotic disorders, gestational DM, and pregestational type 1 or type 2 DM, respectively. We conclude that mothers with chronic diseases, including cardiovascular diseases, hypertension, anemia, genitourinary tract infections, renal diseases, neurotic or psychotic disorders, gestational DM, and pregestational type 1 or type 2 DM, have a significantly higher (*p* < 0.01) prevalence of having offspring with BDs. Mothers with chronic diseases are associated with BDs. It is very important to set up a policy to decrease the prevalence of these maternal chronic diseases; then, we can reduce the incidence of BDs.

## 1. Introduction

Birth defects (BDs) are structural or functional anomalies that occur during intrauterine life and can be identified prenatally, at birth, or later in infancy [1]. Causes and risk factors of BDs include genetics, environmental factors, socioeconomic and demographic factors, infections, and other risk factors [1]. 

Many previous studies have reported the epidemiology and prevalence of BDs [2,3,4,5,6,7,8,9,10]. BDs have been reported to affect approximately 3% of all infants in the United States [4]. The prevalence of BDs is about 2.5% according to data from the European Surveillance of Congenital Anomalies (EUROCAT) study in Europe [11]. It was reported by Chen et al. that the prevalence rate of BDs is 2.72% in Taiwan [12]. BDs are associated with many risk factors, including high or low maternal age [13,14], air pollution [15], teratogenic medications [16,17,18,19,20], pregestational or gestational diabetes [21,22,23,24,25,26,27], chronic diseases [28], genitourinary tract infections [29], and obesity [30]. 

We conducted a study to analyze the association between maternal chronic diseases and BDs and to determine the effect of decreasing maternal chronic diseases on reducing the prevalence of BDs.

## 2. Materials and Methods

### 2.1. Study Population

**Figure 1:**
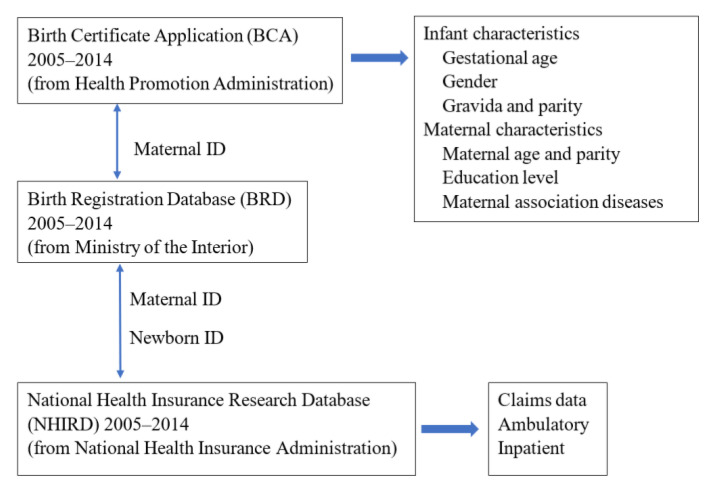
Flow chart of data processing for the three databases.

We linked three databases, including the National Health Insurance Research Database (NHIRD), the Birth Certificate Application (BCA), and the Birth Registration Database (BRD) between 2005 and 2014 in Taiwan. The National Health Insurance program was implemented in 1995; it covers more than 99% of the residents in Taiwan and contracts more than 97% of medical providers. The National Health Insurance Research Database (NHIRD), which contains registration files and reimbursement data, was created for research purposes. The deidentified NHIRD has become one of the best databases in the world for population-based studies, as it includes more than 99% of the population. [31]. These databases cover both public and private hospitals. In order to integrate the infants’ data with the information of their birth mothers, NHIRD data were linked with the BCA and BRD using personal identification numbers and centralized in Taiwan’s Health and Welfare Data Science Center, the details of which can be found in a previous study [32]. Information on children and maternal medical claims of ambulatory and admission care (diagnostic codes, date of visit, prescription, and medical orders) were obtained from the NHIRD. Infants’ information, including birth weight, gestational age, and sex, and mothers’ information, including age; parity; singleton, twin, or triplet; educational status; nationality; and associated diseases were obtained from the BCA and BRD. Figure 1 shows the flow chart of data processing for the three databases.

The birth records (*n* = 2,498,573) between 2005 and 2014 were identified. The children (*n* = 465,569) for whom we were unable to identify the ID number of the mothers from the BCA or BRD were excluded. A total of 2,033,004 newborn infants with complete health insurance, birth, and mothers’ data between 1 January 2005 and 31 December 2014 were included in this study. Codes 740-759 of the International Classification of Diseases 9th Revision-Clinical Modification (ICD-9-CM) were used as the diagnosis of BDs. We followed up every infant for one year. A diagnosis of BDs was made when an infant had a diagnosis with the code 740-759 and at least 2 outpatient visits or 1 admission record.

Ventricular septal defect (VSD), atrial septal defect (ASD), patent ductus arteriosus (PDA), congenital laryngomalacia, and undescended testis were diagnosed when the infants were more than 6 months old. Preterm infants with PDA were excluded from this study because most preterm infants with PDA might close spontaneously later; however, infants more than 6 months old with PDA were included in this study. A total of 55,229 infants were diagnosed with BDs during the study period. Associated maternal diseases were also recorded. 

**Figure 2:**
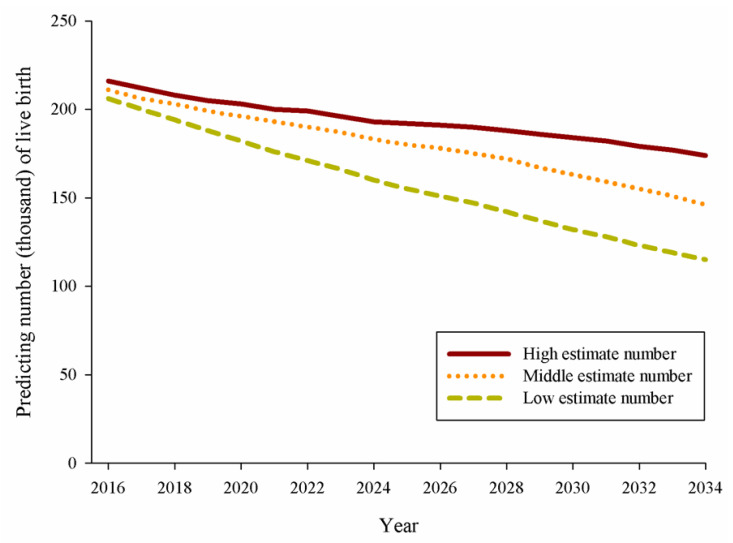
Predicted number of live births from the National Development Council Population Projections in Taiwan, 2016–2034.

We used the report of the National Development Council Population Projections in Taiwan to calculate the high, middle, and low estimates for the population of infants between 2016 and 2034 in Taiwan [33]. Figure 2 shows the high, middle, and low estimates for live births between 2016 and 2034 in Taiwan according to the report of the National Development Council Population Projections for Taiwan. In 2034, the predicted number of live births was 174,000, 146,000, and 115,000 for the high, middle, and low estimates, respectively.

### 2.2. Statistical Analysis

The prevalence of defects was calculated using the method recommended by EUROCAT as follows. The prevalence = the total number of cases divided by the total number of births (live births and stillbirths). The categorical data of the groups were analyzed by a Chi-square test. The crude relative risk (RR) of BDs was defined as the crude prevalence rate in children with maternal disease exposure/crude prevalence rate in children without maternal disease exposure. We used SAS 9.4 software to perform the analysis in this study. A *p*-value < 0.05 was considered to be statistically significant. Population attributable risk percent (PAR%) = (proportion of diseases in population x relative risk (RR) − 1)/(1 + proportion of diseases in population × (RR − 1)). The percentage change in the prevalence of maternal disease in 2034 was compared with the prevalence of maternal disease in 2005–2014. The prevalence of maternal disease was defined as y, and 3 parameters (−1%, −5%, and −10%) of percentage change in maternal disease ((y in 2034 − y in 2005–2014)/y in 2005–2014) was used to estimate the decreased number of newly diagnosed BDs in 2034. The decreased number of newly diagnosed BDs in 2034 by reducing the maternal disease prevalence can be defined as (predicting rate of BDs in 2034) × (1 − (PAR × percentage change in maternal disease)) × (predicting number of live birth in 2034 by high, middle, or low estimation). Institutional review board (IRB) approval was obtained from the ethical committee at Chung Shan Medical University Hospital (CSMUH No: CS17044).

## 3. Results

**Figure 3:**
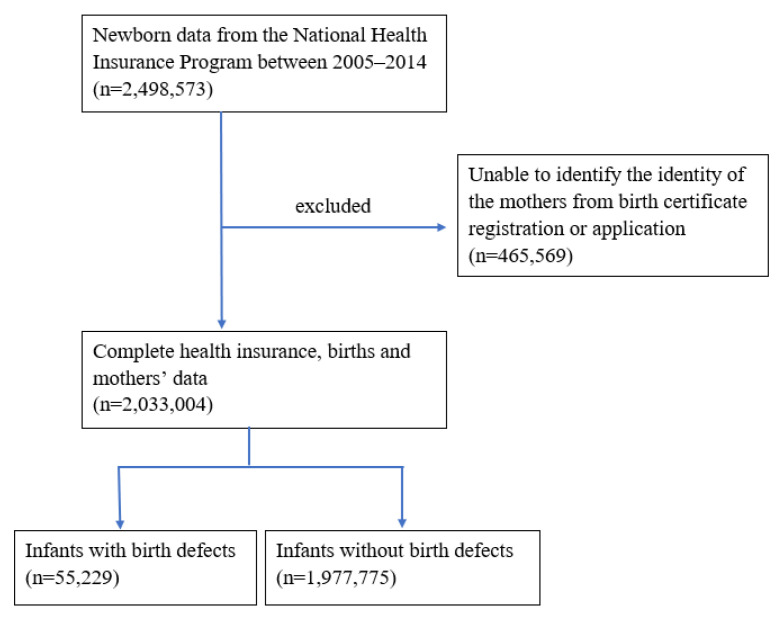
Flow diagram of study cohort.

Figure 3 shows the flow diagram of the study cohort. There were 2,033,004 births and 55,229 infants with BDs diagnosed in Taiwan during the period of 2005 to 2014. The prevalence of BDs was 271.66 (269.4–273.93) per 10,000 births. There was no significant change in the prevalence of BDs from 2005 to 2014 (the change per year was −0.27%, 95% confidence interval = −0.78% to 0.23%, *p* = 0.2819). The prevalence rate of BDs was 86.1 for the cardiovascular system, 57.6 for the genitourinary system, 48.7 for the musculoskeletal system, 25.7 for the digestive system, 21.5 for the respiratory system, 17.8 for cleft palate with or without cleft lip, 14.8 for chromosomal abnormalities, 13.9 for the nervous system, and 6.7 for eye, ear, face, and neck defects per 10,000 births in 2014. VSD and ASD were the most common BDs.

Table 1 shows the prevalence and relative risk of infants with BDs according to maternal diseases. Mothers with cardiovascular diseases, hypertension, anemia, renal diseases, genitourinary infections, neurotic or psychotic disorders, and gestational DM or pregestational type 1 or type 2 DM had a higher relative risk (RR) of BDs (*p* < 0.01).

The relative risk, maternal chronic diseases, and population attributable risk percent (PAR%) of BDs are summarized in Table 2. The effects of preventing maternal chronic diseases resulting in reducing the number of BDs in Taiwan in 2034 are shown in Table 3. If we can decrease the prevalence of hypertension in mothers by 1%, 5%, and 10%, the number of infants with BDs will be reduced by 73, 363, and 725, respectively, in the high-estimate group in 2034. If we can decrease the prevalence of hypertension in mothers by 1%, 5%, and 10%, the number of infants with BDs will be reduced by 60, 302, and 604, respectively, in the middle estimate group in 2034. If we can decrease the prevalence of hypertension in mothers by 1%, 5%, and 10%, the number of infants with birth defects will be reduced by 48, 239, and 479, respectively, in the low estimate group in 2034. The predicted rate of BDs was estimated to be 255.67 per 10,000 births in 2034 in Taiwan.

## 4. Discussion

An estimated 295,000 newborns die within 28 days of birth every year worldwide due to BDs [1]. BDs can contribute to long-term disability, which may have significant impacts on individuals, families, healthcare systems, and societies [1]. This was a 10-year nationwide population-based cohort study. It covered more than 99% of residents in Taiwan and contracted more than 97% of medical providers. We reported that the prevalence of BDs is 2.72% in Taiwan [12], which is nearly the same as in Europe (2.5%) [7] and the United States (3%) [4]. The prevalence reported by the World Health Organization (WHO) is 4–6% [4,7].

Our previous study showed that BDs most commonly involved the cardiovascular system, followed by the genitourinary system, musculoskeletal and skin system, digestive system, respiratory system, cleft palate with or without cleft lip, chromosomal abnormalities, the nervous system, and the eyes, ears, face, and neck. It was reported by Boyd et al. that the most frequently reported BDs were serious cardiac diseases [9]. There was no significant change in the prevalence of BDs during the period from 2005 to 2014 in this study. An upward trend was observed for genitourinary anomalies, and a slight upward trend was observed for cardiovascular anomalies; however, downward trends were observed for anomalies of the nervous, respiratory, digestive, and musculoskeletal systems in this study. Twelve anomaly subgroups increased and five anomaly subgroups decreased from 2003 to 2012 in Europe according to Morries et al. [34]. An increased prevalence of hormone-mediated urogenital anomalies, such as renal agenesis, undescended testis, and hypospadias, was found between 2008 and 2014 in Korea, which were considered to be associated with endocrine factors, as reported by Ko et al. [35]. An increasing trend for cardiovascular anomalies was also found by them, possibly due to the development of diagnostic techniques and more frequent perinatal diagnosis [35]. VSD was the most common BDs in our study, which was consistent with reports from other countries [7,36].

Our data showed that mothers with chronic diseases, including cardiovascular diseases, hypertension, anemia, genitourinary infections, renal diseases, neurotic or psychotic disorders, gestational DM, and pregestational type 1 or type 2 DM, had a significantly higher (*p* < 0.01) prevalence of BDs. The same findings have been reported previously [21,22,23,24,25,26,27,28,29,30]. Mothers with hypertension, DM type 1 or type 2, anemia, congenital heart defects, connective tissue disorders, and mood disorders had an increased overall prevalence of congenital heart disease in their children, as reported by Chou et al. [28]. Feldkamp et at. reported that mothers with genitourinary infections had an increased risk of gastroschisis [29]. Yang et al. reported that diabetic pregnancy was an independent risk factor for major congenital anomalies [25]. Correa et al. reported that pregestational DM was associated with a wide range of BDs [22]. Becerra et al. reported that mothers with insulin-dependent DM, including those with gestational DM who required insulin during the third trimester of pregnancy, had an increased risk of major BDs. Infants of mothers with gestational DM who did not require insulin during pregnancy did not have an increased risk of BDs [24]. Correa et al. reported that pregestational DM was associated with BDs; gestational DM was associated with a limited group of BDs (limited to women with a pregnancy body mass index ≥ 25 kg/m^2^ [22]. Aberg et al. reported that gestational DM was associated with an increased risk of BDs, perhaps due to preexisting but undetected DM type 2 [23].

According to the National Development Council Population Projections for Taiwan [33], we calculated the high, low, and middle estimates for the number of infants (<1 year of age) during 2016 and 2034 in Taiwan. By using the population attributable risk percent (PAR%), our data showed how many BDs could be avoided in 2034 if we decrease the prevalence of maternal chronic diseases by 1%, 5%, and 10% in Taiwan. Our data showed that if we can decrease the prevalence of maternal chronic diseases (including hypertension, cardiovascular diseases, genitourinary tract infections, renal diseases, anemia, neurotic or psychotic disorders, gestational DM, and pregestational DM), then we can reduce the prevalence of BDs. If we can reduce the prevalence of BDs, we may decrease their burden, which may have economic benefits for individuals, families, healthcare systems, and societies. In addition, it is very important to decrease the risk factors, including genetic and environmental factors, which will be more effective in reducing BDs.

There were several limitations to this study. First, we did not perform a prospective study; this was a retrospective cohort study. Second, detailed data for every infant or mother was not obtained in this study. Third, BDs could be caused by mechanisms other than maternal chronic diseases, such as genetic and environmental factors, teratogens, high or low maternal age, maternal obesity, and medications. However, this was a 10-year nationwide cohort study, and the sample size was very large. Finally, we used the crude RR to calculate the population attributable risk percent (PAR%), and this is more efficient for estimating the decrease in the number of BDs by the prevention of maternal disease than the adjusted RR. However, the adjusted relative risk can provide a more accurate estimation of BD prevention, but information on important covariates is not always available in health monitoring systems, especially in developing countries.

## 5. Conclusions

Mothers with chronic diseases, including cardiovascular diseases, hypertension, anemia, renal diseases, genitourinary infections, neurotic or psychotic disorders, gestational DM, and pregestational type 1 or type 2 DM have a higher (*p* < 0.001) prevalence of BDs. Although BDs can be caused by genetic and/or environmental factors, maternal chronic diseases have an association with BDs. Care before, during, and after pregnancy, including healthy nutrition, proper exercise, and avoiding stress and anxiety, can play a useful role in decreasing maternal chronic diseases. The early identification of high-risk pregnant women with such chronic diseases is important. Mothers with such chronic diseases should control their diseases to minimize the risk of BDs.

## Figures and Tables

**Table 1:** Prevalence and relative risk (RR) of infants with birth defects (BDs) according to maternal diseases.

Maternal Diseases	No BDs	BDs	Prevalence Rate (per 10,000 Births)	RR	*p* Value
Cardiovascular diseases	4208 (0.21%)	436 (0.79%)	938.85	3.38	<0.0001
Hypertension	42,648 (2.16%)	2156 (3.90%)	481.21	1.75	<0.0001
Renal diseases	6859 (0.35%)	302 (0.55%)	421.73	1.51	<0.0001
Genitourinary infections	124,044 (6.27%)	4147 (7.51%)	323.50	1.17	<0.0001
Anemia	110,210 (5.57%)	3416 (6.19%)	300.64	1.08	<0.0001
Neurotic and psychotic disorders	54,309 (2.75%)	1689 (3.06%)	301.62	1.08	0.0013
Alcohol-related conditions	1013 (0.05%)	39 (0.07%)	370.72	1.33	0.0767
Drug abuse and dependence	5091 (0.26%)	160 (0.29%)	304.70	1.09	0.2692
DM					
No DM	1,719,174 (86.92%)	47,635 (86.25%)	269.61	Reference	
GDM	237,122 (11.99%)	6866 (12.43%)	281.41	1.04	0.0009
DM (type 1 or type 2)	21,479 (1.09%)	728 (1.32%)	327.82	1.22	<0.0001

RR: relative risk, GDM: gestational diabetes mellitus. DM: diabetes mellitus.

**Table 2:** Prevalence of maternal chronic diseases, relative risk, and PAR% of birth defects between 2005 and 2014.

Maternal Disease	Number of Cases	Prevalence Rate	Relative Risk	PAR%
Hypertension	44,804	2.20%	1.75	1.63%
Cardiovascular disease	4644	0.23%	3.38	0.55%
Renal disease	7161	0.35%	1.51	0.18%
Genitourinary infection	128,191	6.31%	1.17	1.06%
Anemia	113,626	5.59%	1.08	0.45%
Neurotic and psychotic disorders	55,998	2.75%	1.08	0.22%
GDM	243,988	12.00%	1.04	0.48%
DM (type 1 or type 2)	22,207	1.09%	1.22	0.24%

PAR%: population attributable risk percent, GDM: gestational diabetes mellitus. DM: diabetes mellitus.

**Table 3:** The decrease in number of newly diagnosed BDs in 2034 by reducing the prevalence of maternal chronic diseases.

	Percentage Change of the Prevalence of Maternal Disease in 2034 ^†^								
	−1%			−5%			−10%		
Prevalence of Maternal Disease	HE	ME	LE	HE	ME	LE	HE	ME	LE
Hypertension	73	60	48	363	302	239	725	604	479
Cardiovascular disease	25	20	16	123	102	81	245	204	162
Renal diseases	8	6	5	40	33	26	80	67	53
Genitourinary infection	47	39	31	236	196	156	472	393	311
Anemia	20	16	13	100	83	66	201	167	132
Neurotic and psychotic disorders	10	8	6	49	41	32	98	81	64
GDM	22	18	14	107	89	70	214	178	141
Pregestational (Type 1 or type 2) DM	11	9	7	54	44	35	107	89	70

BDs: birth defects; HE: high estimate; ME: middle estimate; LE: low estimate; GDM: gestational diabetes mellitus; DM: diabetes mellitus. ^†^ The percentage change in the prevalence of maternal disease in 2034 was compared with the prevalence of maternal disease in 2005–2014. The prevalence of maternal disease was defined as y, and three parameters (−1%, −5%, and −10%) of percentage change in maternal disease ((y2034 − y2005–2014)/y2005–2014) were used to estimate the decrease in the number of newly diagnosed BDs in 2034. The decreased number of newly diagnosed BDs in 2034 by reducing the maternal disease prevalence can be defined as (predicting rate of BDs in 2034) × (1−(PAR × percentage change in maternal disease × 100)) × (predicting number of live birth in 2034 by high, middle, or low estimation)]. The predicted rate of BDs was estimated to be 255.67 per 10,000 births in 2034. The predicted numbers of live births were 174,000, 146,000, and 115,000 for the high, middle, and low estimates.

## Data Availability

The database is available upon request from the corresponding author under reasonable request.
